# LTD expression is independent of glutamate receptor subtype

**DOI:** 10.3389/fnsyn.2014.00015

**Published:** 2014-07-08

**Authors:** Adam J. Granger, Roger A. Nicoll

**Affiliations:** ^1^Neuroscience Graduate Program, University of California San FranciscoCA, USA; ^2^Department of Cellular and Molecular Pharmacology, University of California San Francisco, San FranciscoCA, USA

**Keywords:** long-term depression, LTD, AMPA receptors, synaptic plasticity

## Abstract

Long-term depression (LTD) is a form of synaptic plasticity that plays a major role in the activity-dependent reshaping of synaptic transmission. LTD is expressed as a decrease in synaptic AMPA receptor number, though the exact mechanism remains controversial. Several lines of evidence have suggested necessary roles for both the GluA1 and GluA2 subunits, and specifically certain interactions with their cytoplasmic tails. However, it is unclear if either GluA1 or GluA2 are absolutely required for LTD. We tested this hypothesis using constitutive knock-outs and single-cell molecular replacement of AMPA receptor subunits in mouse hippocampus. We found that neither GluA1 or GluA2 are required for normal expression of LTD, and indeed a normal decrease in synaptic transmission was observed in cells in which all endogenous AMPA receptors have been replaced by kainate receptors. Thus, LTD does not require removal of specific AMPA receptor subunits, but likely involves a more general modification of the synapse and its ability to anchor a broad range of receptor proteins.

## Introduction

Excitatory synapses in the brain can modify their efficacy to store information in response to specific patterns of activity, either by strengthening through long-term potentiation (LTP) or weakening through long-term depression (LTD; Malenka and Bear, 2004). Both LTP and LTD are expressed through the insertion or removal, respectively, of AMPA-type glutamate receptors, which are heterotetramers comprised of different subunit proteins, GluA1-4 (Kessels and Malinow, 2009). In CA1 pyramidal neurons, where GluA1/GluA2 heteromers dominate (Wenthold et al., 1996; Lu et al., 2009), we have recently demonstrated that LTP is not subunit specific, but can occur with a variety of fast ionotropic glutamate receptors (Granger et al., 2013). In the case of hippocampal LTD, it remains unclear whether either the GluA1 or GluA2 subunits are necessary for synaptic removal and endocytosis of AMPARs.

Previous research has suggested a necessity for both GluA1 and GluA2. GluA2 has been implicated in LTD by studies showing that phosphorylation by PKC at amino acid S880 correlates with increased AMPAR internalization in cultured neurons (Chung et al., 2000), and increased S880 phosphorylation is observed following LTD induction in hippocampal slices (Kim et al., 2001). Additionally, intracellular perfusion of a peptide mimicking the cytoplasmic tail of GluA2 inhibits LTD expression (Kim et al., 2001). Finally, overexpression of a GluA2 S880 phosphomimetic mutant decreased synaptic transmission and occluded LTD, while overexpression of the phosphonull mutant partially blocked LTD expression (Seidenman et al., 2003). However, LTD is intact in GluA2 knockout mice (Meng et al., 2003), indicating that it is not absolutely required. Likewise, GluA1 has been implicated in LTD expression by the finding that the GluA1 S845A knock-in mouse does not express LTD (Lee et al., 2003, 2010), though a recent study found that LTD expression is normal in GluA1 knockout mice (Selcher et al., 2012).

In this study, we directly test whether LTD expression requires specific AMPAR subunit proteins. First, we sought to confirm or deny the necessity of GluA1 or GluA2 using constitutive knock-outs, and found that neither had an effect on LTD expression. To test if any part of the AMPA receptor generally is required for LTD, we used a single-cell molecular replacement technique (Granger et al., 2011, 2013), where all endogenous AMPA receptor subunits are replaced with a foreign kainate-type glutamate receptor. Surprisingly, we found that LTD expression was intact in neurons that entirely lacked AMPA receptors, ruling out their necessity for expression of LTD.

## Materials and methods

### Mouse genetics

Animals were housed according to IACUC guidelines at the University of California, San Francisco. Mice with the *Gria1^fl/fl^*,*Gria2^fl/fl^*, and*Gria3^fl/fl^* (*Gria1-3^fl/fl^*) were generated and genotyped as previously described (Lu et al., 2009).

### *In utero* electroporation

*In-utero* electroporations were performed as previously described (Granger et al., 2013). Briefly, ∼E15.5 pregnant *Gria1-3^fl/fl^* mice were anesthetized with 2.5% isoflurane in O_2_ and injected with buprenorphine for analgesic. Embryos within the uterus were temporarily removed from the abdomen and their left ventricles injected with a 2 μl mixture of 0.5 μg/μl FUGW-Cre:mCherry, 2–3 μg/μl pCAGGS-GluK1-IRES-GFP, and 2–3 μg/μl pCAGGS-Neto2-IRES-mCherry. Embryos were subjected to 50 ms, 35 V pulses five times using tweezer trodes, with the positive electrode placed on the back right hemisphere and the negative electrode on the front left. Following surgery, the electroporated mice were sacrificed on P17-21 for LTD recordings.

### Electrophysiology

Field excitatory post-synaptic potentials (EPSPs) and whole-cell voltage-clamp recordings of CA1 pyramidal neurons were taken from 300 μM acute transverse hippocampal slices cut using a Microslicer ™ DTK-Zero1 (Ted Pella, Inc.). Slices were cut in a chilled high sucrose cutting solution containing (in mM): 2.5 KCl, 7 MgSO_4_, 1.25 NaH_2_PO_4_, 25 NaHCO_3_, 7 glucose, 210 sucrose, 1.3 ascorbic acid, 3 sodium pyruvate. The slices then recovered for 30 min at 34 degrees in artificial cerebral spinal fluid (aCSF) containing (in mM): 119 NaCl, 2.5 KCl, 1 NaH_2_PO_4_, 26.2 NaHCO_3_, and 11 glucose, 2.5 mM CaCl_2_ and 1.3 mM MgSO_4._ The aCSF was bubbled with 95% O_2_ and 5% CO_2_ to maintain pH, and the acute slices allowed to recover at room temperature for 45 min to 1 h. During recording, slices were transferred to a perfusion stage on an Olympus BX51WI upright microscope and perfused at 2.5 ml/min with aCSF containing 0.1 mM pictrotoxin (TCI). 100 μM DL-2-amino-5-phosphonopentanoic acid (APV) (Tocris) was included in the experiments in Figure 2D, and 1 μM (S)-1-(2-amino-2-carboxyethyl)-3-(2-carboxy-5-phenylthiophene-3-yl-methyl)-5-methylpyrimidine-2,4-dione (ACET) (Tocris) in Figures 2C,D. Synaptic responses were evoked by stimulating with a 100 μm tungesten bipolar stimulating electrode (FHC, Inc.) in stratum radiatum of CA1. Simultaneous dual whole-cell recordings were made between GFP- and mCherry-positive experimental cells as identified by epifluorescence, and neighboring non-transfected control cells. Internal recording solution contained (in mM): 135 CsMeSO_4_, 8 NaCl, 10 HEPES, 0.3 EGTA, 5 QX-314, 4 Mg-ATP, 0.3 Na-GTP, and 0.1 spermine. Osmolarity was adjusted to 290–295 mOsm, and pH buffered at 7.3–7.4. AMPAR- and KAR- mediated responses were isolated by clamping the cell at −70 mV, while NMDAR responses were recorded at +40 mV, with amplitudes taken 100 ms following stimulation to avoid contamination by AMPAR current. Field EPSP recordings were made by placing a recording pipette filled with aCSF into stratum radiatum. LTD was induced by stimulating at 1 Hz for 15 min, and voltage-clamping at −40 mV for whole-cell experiments. During whole-cell recordings, membrane holding current, input resistance, and pipette series resistance were monitored. Data was gathered through a MultiClamp 700B amplifier (Axon Instruments), filtered at 2 kHz, digitized at 10 kHz.

**Figure 1:**
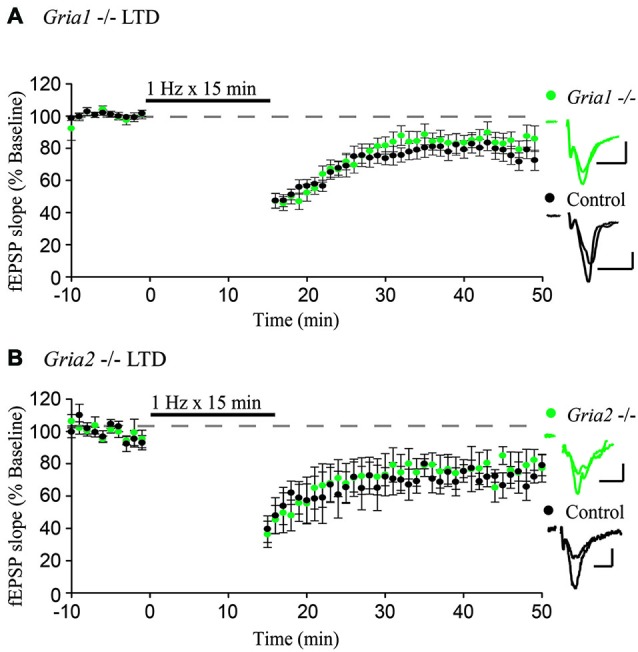
**GluA1 and GluA2 constitutive knockouts demonstrate normal expression of LTD. (A,B)** Field EPSPs recorded from stratum radiatum show normal expression of LTD induced by 1 Hz stimulation for 15 min in *Gria1^−/–^* (*n* = 15, Control *n* = 11) and *Gria2^−/–^* (*n* = 7, Control *n* = 4) hippocampal slices compared to control slices (both *p* > 0.05). Example traces show average field EPSPs from control (black) and knock-out slices (green). Scale bars: 10 ms, 0.5 mV. Error bars represent mean ± s.e.m.

**Figure 2:**
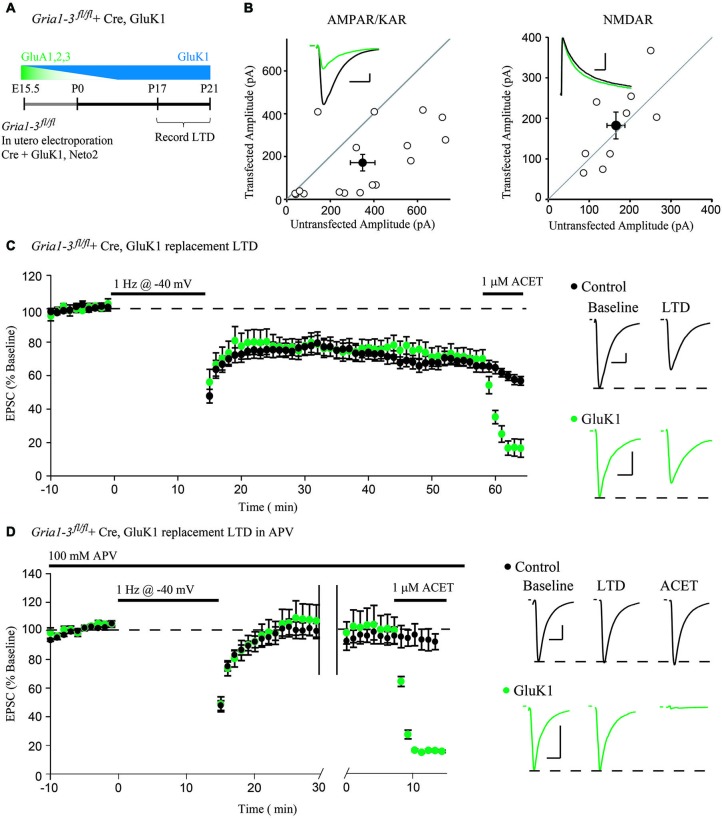
**Molecular replacement of AMPARs with the kainate receptor GluK1 supports normal expression of LTD. (A)** Schematic of the time-course of AMPAR molecular replacement with GluK1. **(B)** Paired whole-cell recordings between Cre + GluK1, Neto2-expressing CA1 neurons and neighboring untransfected control cells in *Gria1-3^fl/fl^* mice show 49% rescue of the EPSC amplitude at −70 mV (AMPA/KAR, *n* = 17, *p* < 0.05) and no change of the NMDAR EPSC at +40 mV (NMDAR, *n* = 9, *p* > 0.05). **(C)** LTD expression is comparable between GluK1 replacement neurons and simultaneously recorded control neurons (*n* = 14, *p* > 0.05 at minute 45). Wash-in of ACET (1 μM) blocks the EPSC in GluK1 replacement neurons but not control. **(D)** LTD expression was blocked in both control neurons and simultaneously recorded GluK1-replacement neurons by 100 μM APV (*n* = 10, *p* > 0.05), and the EPSC is only blocked in GluK1-replacement neurons by wash-in of ACET. Example traces show averaged EPSCs from GluK1 replacement (green) and control (black) neurons both before LTD induction (Baseline) and after 45 min (LTD), and after wash-in of ACET. Scale bars: 20 ms, 100 pA for AMPAR/KARs, 100 ms for NMDARs. Error bars represent mean ± s.e.m.

### Statistics

Statistical comparisons were made using a Mann-Whitney U test. For all field LTD experiments, comparisons were made 45 min following the beginning of induction of LTD between interleaved slices from genetic deletion mice and wild-type littermate controls. For all molecular replacement experiments, comparisons were made between paired replacement and non-transfected control neurons. If one of the paired cells was lost during the course of the recording, the data from the remaining cell would be kept. Comparisons were made 45 min following induction of LTD, and the reported n-values represent the number of cells at that time point. Data analysis was carried out in Igor Pro (Wavemetrics), Excel (Microsoft), and R (The R Project for Statistical Computing^1^).

## Results

To test whether GluA1 or GluA2 are specifically required for LTD, we first used constitutive deletions of GluA1 and GluA2, respectively, which constitute the majority of synaptic AMPARs in CA1 pyramidal neurons (Wenthold et al., 1996; Lu et al., 2009). We recorded from acute hippocampal slices taken from GluA1 and GluA2 constitutive knockout mice (*Gria1^−/–^* and *Gria2^−/–^*), with slices from wild-type littermates used as a control. After obtaining a stable field EPSP baseline from stratum radiatum, LTD was induced by stimulating at a low frequency (1 Hz) for 15 min. In agreement with previous findings (Selcher et al., 2012), LTD expression was intact in GluA1 knockout slices, indistinguishable from LTD recorded from control slices (Figure 1A). This indicates that the GluA1 subunit is not required for expression of LTD. Similar results were also observed in slices taken from GluA2 constitutive knockout mice (Figure 1B), also in agreement with previously published results (Meng et al., 2003). Combined, these results argue strongly against a specific requirement for any individual AMPAR subunit.

However, the possibility remains that either GluA1 or GluA2 can mediate the activity-dependent removal of AMPARs during LTD. To address this possibility, we turned to a single-cell molecular replacement strategy (Granger et al., 2011), where all endogenous AMPARs are substituted for kainate-type glutamate receptors. Kainate receptors (KARs) are a separate class of fast, ionotropic glutamate receptor not normally found at CA1 synapses that share little sequence homology with AMPARs (Contractor et al., 2011). The advantage to using KARs is that it allows us to assay synaptic transmission in the absence of AMPARs. Additionally, KARs may be used as a functional null receptor with which we can perform domain-swapping experiments with AMPARs (Lu et al., 2010) to narrow down regions necessary for LTD. To achieve molecular replacement, we co-transfected Cre into CA1 neurons of *Gria1-3^fl/fl^* mice by *in utero* electroporation (Figure 2A), which results in complete loss of endogenous AMPA receptors by P10 (Granger et al., 2013), along with a replacement GluK1 subunit and Neto2, a KAR auxiliary subunit (Tomita and Castillo, 2012). This results in a sparse pattern of transfection, ensuring that the hippocampus is predominantly wild-type. Three weeks following the transfection, we recorded from GluK1-replacement CA1 neurons paired with neighboring untransfected control neurons. We found that GluK1 replacement rescued approximately 49% of the control EPSC amplitude, and had no effect on the NMDAR EPSC (Figure 2B), in line with previous findings (Granger et al., 2013). After recording a stable baseline EPSC amplitude for 10 min, LTD was induced by stimulating at 1 Hz for 15 min while depolarizing both neurons to −40 mV. Surprisingly, we observed comparable expression of LTD between the GluK1 replacement neuron and control (Figure 2C), indicating that the LTD can be expressed independent of AMPARs. Wash-in of ACET, a highly selective GluK1-antagonist, confirmed that the replacement neuron expressed only GluK1 (Figure 2C). To ensure that the LTD expressed in GluK1 replacement neurons used the same mechanism as control neurons, we tried expressing LTD in the presence of the NMDAR antagonist APV. In both GluK1 and control neurons, LTD expression was blocked by APV, though only the replacement neuron was affected by wash-in of ACET (Figure 2D). Based on these results, NMDAR-dependent LTD does not require AMPARs, but can be expressed with alternative ionotropic glutamate receptors.

## Discussion

Our results suggest a model of LTD whereby specific modifications to individual glutamate receptor proteins are not required for the activity-dependent removal of synaptic receptors. These data are difficult to reconcile with the findings that LTD is impaired by overexpressing GluA2 with mutations to the S880 phosphorylation site (Seidenman et al., 2003) and blocked by germline mutations of the S845 site on GluA1 (Lee et al., 2003, 2010). One possibility is that these mutations have modulatory effects on synaptic transmission that affect baseline trafficking of the receptor, which could interfere with synaptic plasticity. For example, the PKA S845A mutation has been shown to impair CaMKII-mediated incorporation of GluA1 into the synapse (Esteban et al., 2003), which might occlude LTD. Indeed, in a previous study we found that the requirement of GluA1 for LTP is secondary to its requirement in forming an adequate reserve pool of AMPARs, since replenishing the reserve pool with an alternate glutamate receptor also rescued LTP (Granger et al., 2013). It is therefore possible that these mutations are affecting some aspect of baseline trafficking that occludes or interferes with LTD. Another possibility is that both receptor-specific and receptor-independent mechanisms are involved in LTD expression. Therefore, one could argue that both GluA1 and GluA2 undergo a unique modification to promote receptor endoctyosis during LTD, and interfering with these modifications with targeted knock-ins or dominant negatives may impair LTD even though complete deletion leaves LTD intact. However, the presence of fully normal levels of LTD even after molecular replacement with GluK1 argues against this possibility. Given the lack of sequence homology between GluK1 and the AMPAR subunits, any AMPAR-specific modifications are unlikely to be preserved.

The present results on LTD, along with our recent results on LTP (Granger et al., 2013), indicate that the trapping and untrapping of receptors at synapses is independent of any highly targeted and specific modifications to the AMPAR cytoplasmic tail. Instead, LTD likely involves a broader reorganization of the synapse as a whole that can affect a diverse range of proteins. In fact, electrical and chemical induction of LTD does correlate with a physical shrinking of dendritic spines (Zhou et al., 2004), although in certain conditions a decrease in spine volume can be dissociated from a decrease in synaptic transmission (Wang et al., 2007; He et al., 2011). This LTD-induced shrinking of dendritic spines has been linked to destabilization of actin (Okamoto et al., 2004), and inhibitors of actin depolymerization also block expression of LTD (Wang et al., 2007). Whether or not LTD produces a visible morphological change, such actin depolymerization may impair the integrity of the structural scaffold that anchors AMPARs, or in the case of our experiments, KARs, in the post-synaptic density, resulting in a decrease in synaptic transmission. This raises the possibility that specific protein-protein interactions are not involved in plasticity, and it has been speculated that molecular crowding within the dense thicket of proteins in the postsynaptic density (PSD) might control the acquisition and loss of receptors (Renner et al., 2009; Santamaria et al., 2010). Perhaps the actin cytoskeleton, which is known to play a critical role in activity-dependent changes in spine volume, also controls the physical properties of the PSD.

## Author Contributions

Adam J. Granger designed the study, collected and analyzed data, and wrote the paper. Roger A. Nicoll conceived of the study and wrote the paper. All authors discussed the results and commented on the manuscript.

## Conflict of interest statement

The authors declare that the research was conducted in the absence of any commercial or financial relationships that could be construed as a potential conflict of interest.

## Footnotes

^1^http://www.r-project.org/
